# Diabetes mellitus and prostate cancer risk among older men: population-based case–control study

**DOI:** 10.1038/sj.bjc.6601857

**Published:** 2004-05-04

**Authors:** A L Coker, M Sanderson, W Zheng, M K Fadden

**Affiliations:** 1 University of Texas-Houston School of Public Health, Houston, TX 77225, USA; 2 University of Texas-Houston School of Public Health at Brownsville, Brownsville, TX 78520, USA; 3 Center for Health Services Research and Vanderbilt-Ingram Cancer Center, Vanderbilt University, Nashville, TN 37232-8300, USA

**Keywords:** prostate neoplasms, epidemiologic studies, diabetes mellitus, African-American

## Abstract

We investigate the relation between diabetes mellitus and risk of prostate cancer among older (age 65–79 years) men in a population-based case–control study of 407 incident histologically confirmed cases registered in the South Carolina Central Cancer Registry between 1999 and 2001 (70.6% response rate); controls were 393 men identified through the Health Care Financing Administration Medicare beneficiary file for South Carolina in 1999 (63.8% response rate). After adjusting for age, race, and prostate cancer screening in the past 5 years, a history of diabetes mellitus was associated with a reduced risk of prostate cancer (adjusted odds ratio (aOR)=0.64; 95% confidence interval (CI)=0.45, 0.91). The protective effect was stronger for those with complications associated with diabetes (aOR=0.61; 95% CI=0.42, 0.90) and for African-American men (aOR=0.36; 95% CI=0.21, 0.62). Additional research is needed to understand the biologic mechanisms by which diabetes may influence prostate cancer risk; genetic factors may play an important role in understanding this association.

Four prospective studies (Thompson *et al*, 1989; Adami *et al*, 1991; Giovannucci *et al*, 1998; Weiderpass *et al*, 2002) and one hospital-based case–control study (Rosenberg *et al*, 2002) have found a reduced risk of prostate cancer associated with type II diabetes mellitus. The cohort studies suggest that risk of prostate cancer decreases with increasing time since diabetes diagnosis. Detection bias, potentially introduced if those with diabetes are more likely to receive prostate cancer screening, does not appear to explain the association (Giovannucci, 2001). These findings contrast with three additional prospective (Coughlin *et al*, 1996; Will *et al*, 1999; Gapstur *et al*, 2001) and three case–control studies (Mishina *et al*, 1985; LaVecchia *et al*, 1994; Tavani *et al*, 2002) that did not find an association between diabetes and prostate cancer.

Giovannucci (2001) suggested that future studies investigate: (1) the time lag between diabetes mellitus diagnosis and subsequent prostate cancer; (2) diabetes complications and prostate cancer risk; and (3) any association between diabetes and stage of prostate cancer at detection. The purpose of this analysis was to investigate the association between diabetes mellitus and prostate cancer among African-American and Caucasian men in a population-based case–control study.

## MATERIALS AND METHODS

Details of this population-based case–control study have been reported elsewhere (Sanderson *et al*, 2004). Briefly, cases aged 65–79 years diagnosed with primary invasive histologically confirmed prostate cancer between October 1999 and September 2001 were identified through the South Carolina Central Cancer Registry (SCCCR). During the study period, a total of 551 Caucasian men and 245 African-American men with localised disease (stages I and II), and 98 Caucasian men and 70 African-American men with advanced disease (stages III and IV) reported to the SCCCR were eligible. All eligible cases with advanced disease, and a random sample of men with localised disease within 5-year age groups (42% of Caucasian cases and 83% or African-American cases) were selected. A total of 426 prostate cancer cases (70.6% of eligible cases) completed a standardised telephone interview. Of potentially eligible cases, 71 refused (11.8%), 24 died prior to the interview (4.0%), 59 were not located (9.8%), and 23 were too sick to participate (3.8%). After eliminating seven prevalent prostate cancer cases and 11 cases who did not provide complete interview data, 407 cases remained for analyses.

Control subjects were South Carolina residents aged 65–79 years randomly sampled from the 1999 Health Care Financing Administration (HCFA) Medicare beneficiary file. They were frequency matched to cases on age (5-year age groups), race (Caucasian, African-American), and geographical region (western, middle and eastern third of the state). A total of 482 control subjects (63.8%) completed the interview. Of potentially eligible controls, 108 refused (14.3%), 22 died prior to the interview (2.9%), 112 were not located (14.8%), and 32 were too sick to participate (4.2%). After eliminating 52 controls with prevalent prostate cancer and 37 controls whose interviews were incomplete, 393 controls remained for analysis.

Cases and controls were recruited through mailings that described the study and informed the potential participant that an interviewer would call them soon. Since the HCFA file does not contain telephone numbers, controls whose phone numbers could not be located through directory assistance, telephone, or reverse directories were sent an additional letter asking for a preferred contact number. Trained interviewers from the University of South Carolina Survey Research Laboratory conducted computer-assisted telephone interviews with subjects who provided verbal consent with the understanding that written consent would be obtained. Telephone interviews of 30–40 min in length collected information on demographic characteristics, socioeconomic status, alcohol and tobacco use, physical activity, diet, medical history (including diabetes, stroke, myocardial infarction, cirrhosis or other liver disease, hypertension, and hypercholesterolemia), family history of cancer, history of sexually transmitted diseases, and farm-related work activities and exposures. Men were specifically asked if they had ever been diagnosed with diabetes mellitus (also known as high blood sugar or sugar diabetes). Most exposures pertained to the period prior to a reference date, the date of diagnosis for cases and an assigned date for controls. Institutional Review Boards of the University of South Carolina, the Centres for Disease Control and Prevention, and the National Cancer Institute approved this project's data collection procedures.

We used unconditional logistic regression to estimate the odds ratio of prostate cancer associated with a prior history of diabetes mellitus while controlling for potential confounding factors (Breslow and Day, 1980). The latter included age, race, educational level, annual income, occupation, marital status, family history of prostate cancer, body mass index, diet, physical activity, alcohol and tobacco use, and number of prostate cancer screenings (digital rectal exam (DRE) or prostate-specific antigen (PSA) in the past 5 years prior to the reference date. As screening by DRE and PSA were highly correlated (*r*=0.61, *P*<0.0001), we created a variable to combine the number of prostate cancer screenings in the past 5 years by DRE or PSA. Further because diabetic men may be more frequently screened by their physicians and for diabetes, we included prostate cancer screening in logistic models. Body mass index, defined as self-reported weight (kg) before reference date divided by the square of self-reported height (m^2^), was included as a continuous variable in logistic regression models and categorised as normal weight (<25.0 kg m^−2^), overweight (25.0–29.9 kg m^−2^), or obese (⩾30.0 kg m^−2^).

Diet, assessed in a 20-item food frequency questionnaire adapted from Hayes *et al* (1999), covered foods consumed at least once a year in the period prior to the reference date. The animal fat food group included: eggs, whole milk, cheese, ice cream, beef, stew, mixed meat dishes, hot dogs, luncheon meats, bacon, other pork, liver, and chicken; the lycopene food group included: raw tomatoes, cooked tomatoes, and watermelon. Servings per week of food groups were categorised into quartiles among controls. Engaging in strenuous or moderate leisure-time physical activity for an average of one or more hours a week since age 18 years were categorised as tertiles within the active group.

The primary exposure, diabetes mellitus, was included as a dichotomous variable (yes/no history of diabetes). As it has been suggested that diabetes complications may be more strongly associated with prostate cancer risk (Weiderpass *et al*, 2002), we created a variable to indicate severe diabetes complications that combined reported diabetes with hypertension, hypercholesterolemia, stroke, or a myocardial infarction. Both, such men and those with no such complications were compared to those without diabetes.

## RESULTS

The final sample included 407 prostate cancer cases (166 African-American and 241 Caucasian men) and 393 controls (166 African-American and 227 Caucasian men). Owing to frequency matching, cases and controls were, in general, comparable in age and race crude odds ratios (ORs) and 95% confidence intervals (CIs) for prostate cancer are presented Tables 1

**Table 1:** Comparison of cases and controls for demographic and other risk factors for prostate cancer

	**Cases (*n*=407)**	**Controls (*n*=393)**
**Risk factor**	***N* (%)**	***N* (%)**
*Age (years)*^a^		
65–69	192 (46.2%)	186 (43.4%)
70–74	134 (32.2%)	125 (29.1%)
75–79	90 (21.6%)	118 (27.5%)
		
*Race*^a^		
African-American	171 (41.1%)	171 (39.9%)
Caucasian	245 (58.9%)	258 (60.1%)
		
*Education*^a^		
Less than 8th grade	106 (25.7%)	89 (20.8%)
<High school graduate	55 (13.4%)	69 (16.1%)
High school graduate	101 (24.5%)	102 (23.8%)
Some college or technical school	54 (13.1%)	77 (18.0%)
College graduate	96 (23.3%)	92 (21.5%)
Missing	4	0
*P*-value for trend	0.41	
		
*Annual income*		
<$20 000	122 (34.4%)	106 (32.0%)
$20 000–$29 999	72 (20.3%)	58 (17.5%)
$30 000–$39 999	42 (11.8%)	54 (16.3%)
$40 000–$49 999	25 (7.0%)	36 (10.9%)
$50 000 or more	94 (26.5%)	77 (23.3%)
Missing	52	62
*P*-value for trend	0.69	
		
*Marital status*		
Single/separated/divorced/widowed	73 (18.2%)	80 (20.6%)
Married/living as married	328 (81.8%)	308 (79.4%)
Missing	6	5
		
*BMI* (*kg m*^−*2*^)		
Normal weight (⩽24.9 BMI)	105 (26.5%)	113 (29.5%)
Overweight (25.0–29.9 BMI)	198 (50.0%)	175 (45.7%)
Obese (⩾30.0 BMI)	93 (23.5%)	95 (24.8%)
Missing	11	10
*P*-value for trend	0.75	
		
*Family history of prostate cancer*		
Yes	120 (30.2%)*	60 (15.4%)
No	278 (69.8%)	329 (85.6%)
Missing	9	4
		
*History of benign prostatic hyperplasia*		
Yes	159 (39.7%)*	102 (26.2%)
No	242 (60.3%)	288 (73.8%)
Missing	6	3
		
*History of high cholesterol*		
Yes	122 (30.6%)**	147 (38.2%)
No	277 (69.4%)	238 (61.8%)
Missing	8	8
		
*History of hypertension*		
Yes	210 (52.2%)	206 (53.4%)
No	192 (47.8%)	180 (46.6%)
Missing	5	4
		
*Prostate cancer screening (#of PSAs or DREs in the past 5 years)*^a^		
0	30 (7.4%)	57 (13.7%)
1–2	44 (10.9%)	57 (13.7%)
3–4	52 (12.9%)	66 (15.9%)
5–6	73 (18.1%)	83 (20.0%)
7–8	52 (12.9%)	49 (11.8%)
9–10	152 (37.7%)	104 (25.0%)
Missing	13	13
*P*-value for trend	<0.0001	
		
*Drinking duration (years)*		
0	130 (33.7%)	110 (30.2%)
<25	75 (19.4%)	74 (20.3%)
25–45	94 (24.4%)	87 (23.9%)
>45	87 (22.5%)	93 (25.6%)
Missing	21	29
*P*-value or trend	0.29	
		
*Smoking duration (years)*		
0	106 (26.5%)	116 (30.1%)
<25	116 (29.0%)	109 (28.3%)
25–45	103 (25.8%)	93 (24.2%)
>45	75 (18.8%)	67 (17.4%)
Missing	7	8
*P*-value or trend	0.30	
		
*Consumption of animal fat (quartiles)*		
⩽12.7	102 (28.3%)	85 (25.0%)
12.8–18.95	82 (22.7%)	85 (25.0%)
18.96–26.7	92 (25.8%)	83 (24.4%)
⩾26.8	85 (23.6%)	87 (25.0%)
Missing	46	53
*P*-value or trend	0.46	
		
*Consumption of lycopene (quartiles)*		
⩽2.6	98 (26.7%)	86 (24.6%)
2.7–4.5	97 (26.4%)	85 (24.4%)
4.6–8.0	96 (26.2%)	80 (22.9%)
⩾8.1	76 (20.7%)	98 (28.1%)
Missing	40	44
*P*-value for trend	0.11	
		
*Strenuous or moderate physical activity (tertiles among active)*		
None	67 (17.9%)	69 (19.6%)
<4.0	53 (14.2%)	52 (14.8%)
4.0–12	109 (29.1%)	109 (31.0%)
>12	145 (38.8%)	122 (34.7%)
Missing	33	41
*P*-value for trend	0.33	

* *P*<0.01;

** *P*=0.01–0.05.

BMI=body mass index; PSA=prostate-specific antigen; DRE=digital rectal exam.

, 2

**Table 2:** OR for prostate cancer and diabetes among men aged 65–79 years

	**Case, *N*=407**	**Control, *N*=393**	**Adjusted^a^ OR (95% CI)**
*History of diabetes*			
Missing	7	4	
No	330 (82.1%)	289 (74.3%)	1.00 (REF)
Yes	72 (17.9%)	100 (25.7%)	0.64 (0.45, 0.91)
No complications	14 (3.5%)	17 (4.4%)	0.79 (0.37, 1.67)
With complications	58 (14.4%)	83 (21.3%)	0.61 (0.42, 0.90)

a Adjusted for age (categorical variable), race (African-American compared with Whites), South Carolina region (three areas), and prostate cancer screening in the past 5 years before the referent date (yes annual screening *vs* no).

OR=odds ratios; CI=confidence interval.

and 3

**Table 3:** ORs for prostate cancer and diabetes among men aged 65–79 years by risk factor strata

	**History of diabetes**		
	**NoCase/control**	**YesCase/control**	**aOR^a^ (95% CI) for diabetes and prostate cancer by strata**
			
*Stage at prostate cancer diagnosis*			
Local	254/289	48/100	0.54 (0.36, 0.80)
Advanced	76/289	24/100	1.00 (0.58, 1.70)
			
*Prostate cancer screening (PSA and DRE annually)*^a^			
Less than annual	133/152	29/54	0.61 (0.36, 1.01)
Annual^b^	186/131	54/42	0.68 (0.41, 1.12)
			
*Age at diagnosis (years)*			
65–69	152/120	35/50	0.59 (0.36, 0.99)
70–74	104/84	25/30	0.65 (0.34, 1.24)
75–79	74/85	12/20	0.72 (0.33, 1.60)
			
*Race*			
Caucasian	200/190	40/36	1.08 (0.66, 1.78)
African-American	130/99	32/64	0.36 (0.21, 0.62)
			
*Family history of prostate cancer*			
No	230/247	48/80	0.64 (0.43, 0.98)
Yes	98/40	22/20	0.47 (0.22, 1.00)
			
*History of benign prostatic hyperplasia*			
No	201/204	37/80	0.46 (0.29, 0.73)
Yes	124/82	35/20	1.12 (0.60, 2.10)
			
*BMI* (*kg m*^−*2*^)			
Normal weight (⩽24.9 BMI)	96/98	9/15	0.58 (0.33, 1.01)
Overweight (25.0–29.9 BMI)	169/134	29/41	0.62 (0.33, 1.15)
Obese (⩾30.0 MI)	61/52	32/42	0.62 (0.33, 1.15)

a Adjusted for age (categorical variable), race (African-American compared with Whites), South Carolina region (three areas), and prostate cancer screening in the past 5 years before the referent date (yes annual screening *vs* no).

b Annual defined as PSA or DRE at least once a year for the past 5 years.

OR=odds ratios; aOR=adjusted odds ratio; CI=confidence interval; BMI=body mass index; PSA=prostate-specific antigen; DRE=digital rectal exam.

for the risk factors of interest. Having had the benign prostatic hyperplasia (BPH) or a family history of prostate cancer was associated with incident prostate cancer as were annual PSA tests or DRE over the years prior to the referent date (*P*-value for trend <0.0001). A history of high cholesterol was associated with reduced prostate cancer risk (adjusted OR (aOR)=0.72; 95% CI=0.53, 0.96). No other risk factors were associated with prostate cancer risk in these data.

Presented in Table 2 are the multivariate odds ratios for diabetes and prostate cancer risk. Our three frequency matching variables (age, race, South Carolina region) were included in multivariate logistic regression models as confounders as was prostate cancer screening in the past 5 years to minimise detection bias No other confounding factor materially affected the OR for diabetes and prostate cancer. After adjustment, diabetes was associated with a reduced risk of prostate cancer (aOR=0.64; 95% CI=0.45, 0.91) more marked among those with complications.

Table 3 presents the association between diabetes and prostate cancer among selected prostate cancer risk factor strata. In general, the reduced risk of prostate cancer associated with diabetes was statistically significant for those with localised disease and among younger men (aged 65–69 years), African-American men, men without a family history of prostate cancer, and those with no history of BPH.

## DISCUSSION

Our findings are consistent with four prospective studies (Thompson *et al*, 1989; Adami *et al*, 1991; Giovannucci *et al*, 1998; Weiderpass *et al*, 2002) and one hospital-based case–control study (Rosenberg *et al*, 2002) that reported a reduced risk of prostate cancer associated with diabetes mellitus. We adjusted for prostate cancer screening to reduce detection bias since diagnosed diabetics would typically be screened more frequently than nondiabetics. Our findings concur with others (Giovannucci, 2001) that detection bias does not appear to explain the reduced risk of prostate cancer associated with diabetes. When we used the same definition of later stages (II–IV) as in a New York hospital-based study (Rosenberg *et al*, 2002), our results (aOR 0.6; 95% CI=0.5, 0.9) are consistent with those reported (0.5; 95% CI=0.2–0.9) for diabetes and prostate cancer. Our finding that this risk was present only among African-American men also contrasts with a previous report (Rosenberg *et al*, 2002) that Hispanic and Caucasian men, yet not African-American men, were at reduced risk of prostate cancer. Our finding of a stronger association among those with diabetic complications is consistent with a large cohort study (Weiderpass *et al*, 2002).

It is plausible that diabetes mellitus might increase or reduce the risk of subsequent prostate cancer incidence based on different biologic mechanisms. Diabetes results in higher circulating insulin and glucose levels and perhaps higher free insulin-like growth factor I (Giovannucci, 2001), which may be growth enhancing and therefore increase prostate cancer risk. While hyperglycemia may be growth enhancing, androgen levels are lower in severe diabetes (Ando *et al*, 1984) probably due to a toxic effect of hyperglycemia on the Leydig cells of the testis.

Our study has several limitations. We do not have data on the age at first diabetes diagnosis and so cannot confirm reports that diabetes diagnosed more than 10 years prior to prostate cancer shows a stronger association (Giovannucci *et al*, 1998; Tavani *et al*, 2002). Given the age range of subjects (65–79 years) and the mean age of diabetes diagnosis in the USA of 46.5 years (http://www.cdc.gov/diabetes/st atistics), it is likely that the majority of subjects reporting diabetes had had this condition for at least 10 years. Diabetes was self-reported and we do not have the data to confirm the diagnosis. However, the prevalence among Caucasian men aged 65–79 years reported in this study (16%) was comparable to that reported by South Carolina in 2000 as part of a Centers for Disease Control (CDC) diabetes prevalence study (15.4%) (Centers for Disease Control and Prevention, 2000). Similarly, our diabetes prevalence of 29% among older African-American men is consistent with CDC prevalence data (http://www.cdc.gov/diabetes/st atistics/prev/state). Other limitations include a lower response rate among African-Americans than Caucasians. The refusal rates did not differ by race, but the proportion that could not be located was higher among African-American (19.3%) than Caucasian (6%) cases and controls. Another source of misclassification was the memory problems common among older men; this misclassification was likely nondifferential, thereby reducing our ability to identify weak associations.

This is the first population-based case–control study to address diabetes and prostate cancer risk among both African-American and Caucasian men. Few studies have included sufficient numbers of African-American men (Rosenberg *et al*, 2002) to determine whether they are at reduced risk of prostate cancer if they have a history of diabetes. Hospital- and clinic-based studies of the matter may bias the OR toward a protective effect, since such controls may be more likely to have diabetes or have its complications. Our finding of a reduced risk of diabetes in a population-based study provides additional evidence that the association is not due to such a selection bias. South Carolina has had one of the highest incidence rates of prostate cancer (US Cancer Statistics Working Group, 2002) and African-American men are at significantly greater risk than their Caucasian counterparts (Ries *et al*, 2003). Genetic factors that may differ markedly by race and ethnicity may be an explanation for our finding of a protective effect only in African-American men. Other explanations include racial differences in health care. An understanding of this association may be helpful in prostate cancer prevention efforts.

## References

[bib1] Adami HO, McLaughlin J, Ekbom A (1991) Cancer risk in patients with diabetes mellitus. Cancer Causes Control 2: 307–314 1932543 10.1007/BF00051670

[bib2] Ando S, Rubens R, Rottiers R (1984) Androgen plasma levels in male diabetes. J Endocrinol Invest 7: 21–24 6232308 10.1007/BF03348370

[bib3] Breslow NE, Day NE (1980) Statistical Methods in Cancer Research, Vol. 1, The Analysis of Case–Control Studies. Lyon, France: IARC 7216345

[bib4] Centers for Disease Control and Prevention, National Center for Chronic Disease Prevention and Health Promotion, Division of Adult and Community Health. Division of Adult and Community Health (2000) Behavioral Risk Factor Surveillance System. Atlanta, GA: CDC Division of Diabetes Translation

[bib5] Coughlin SS, Neaton JD, Sengupta A (1996) Cigarette smoking as a predictor of death from prostate cancer in 348,874 men screened for the Multiple Risk Factors Intervention Trial. Am J Epidemiol 143: 1002–1006 8629606 10.1093/oxfordjournals.aje.a008663

[bib6] Gapstur SM, Gann PH, Colangelo LA, Barron-Simpson R, Kopp P, Dyer A, Liu K (2001) Postload plasma glucose concentration and 27-year prostate cancer mortality. Cancer Causes Control 12: 763–772 11562117 10.1023/a:1011279907108

[bib7] Giovannucci E (2001) Medical history and etiology of prostate cancer. Epidemiol Rev 23: 159–162 11588842 10.1093/oxfordjournals.epirev.a000783

[bib8] Giovannucci E, Rimm EB, Stampfer MJ (1998) Diabetes mellitus and risk of prostate cancer (United States). Cancer Causes Control 9: 3–9 9486458 10.1023/a:1008822917449

[bib9] Hayes RB, Ziegler RG, Gridley G, Swanson C, Greenberg RS, Swanson GM, Schoenberg JB, Silverman DT, Brown LM, Pottern LM, Liff J, Schwartz AG, Fraumeni Jr JF, Hoover RN (1999) Dietary factors and risks for prostate cancer among blacks and whites in the United States. Cancer Epidemiol Biomark Prev 8: 25–34 9950236

[bib10] LaVecchia C, Negri E, Franceschi E (1994) A case–control study of diabetes mellitus and cancer risk. Br J Cancer 70: 950–953 7947103 10.1038/bjc.1994.427 PMC2033532

[bib11] Mishina T, Watanabe H, Araki H (1985) Epidemiological study of prostate cancer by matched-pair analysis. Prostate 6: 423–436 4088954 10.1002/pros.2990060411

[bib12] Ries LAG, Eisner MP, Kosary CL, Hankey BF, Miller BA, Clegg L, Mariotto A, Fay MP, Feuer EJ, Edwards BK (2003) SEER Cancer Statistics Review, 1975–2000. Bethesda, MD: National Cancer Institute

[bib13] Rosenberg DJ, Neugut AI, Ahsan H, Shea S (2002) Diabetes mellitus and the risk of prostate cancer. Cancer Invest 20: 157–165 11901534 10.1081/cnv-120001141

[bib14] Sanderson M, Coker AL, Logan P, Zheng W, Fadden MK (2004) Lifestyle and prostate cancer among older African-American and Caucasian men in South Carolina. Cancer Causes Control, in press 10.1023/B:CACO.0000036172.63845.d4 PMC5521001 15280622

[bib15] Tavani A, Gallus S, Bosetti C, Tzonou A, Lagiou P, Negri E, Trichopoulos D, LaVecchia C (2002) Diabetes and risk of prostate cancer. Eur J Cancer Prev 11: 125–128 11984129 10.1097/00008469-200204000-00003

[bib16] Thompson MM, Garland C, Barrett-Connor E (1989) Heart disease risk factors, diabetes, and prostatic cancer in an adult community. Am J Epidemiol 129: 511–517 2916544 10.1093/oxfordjournals.aje.a115162

[bib17] US Cancer Statistics Working Group (2002) United States Cancer Statistics: 1999 Incidence. Atlanta, GA: Department of Health and Human Services, Centers for Disease Control and Prevention and National Cancer Institute

[bib18] Weiderpass E, Ye W, Vainio H, Kaaks R, Adami HO (2002) Reduced risk of prostate cancer among patients with prostate cancer. Int J Cancer 102: 258–261 12397646 10.1002/ijc.10685

[bib19] Will JC, Vinicor F, Calle E (1999) Is diabetes mellitus associated with prostate cancer incidence and survival? Epidemiology 10: 313–318 10230844

